# Failure behavior of zirconia crowns subjected to air abrasion with different particle sizes

**DOI:** 10.1590/0103-6440202304998

**Published:** 2023-03-06

**Authors:** João Cézar Mosele, Andressa Restani Oliveira, Gabriela Pizzolatto, Paula Benetti, Marcia Borba

**Affiliations:** 1 Graduate Program in Dentistry, Dental School, University of Passo Fundo(UPF), Passo Fundo, RS, Brazil.

**Keywords:** Ceramics, air abrasion, computer-aided design

## Abstract

This study aimed to investigate the failure behavior of 3 mol.% yttria-stabilized tetragonal zirconia polycrystal (3Y-TZP) prosthetic crowns air-abraded with aluminum oxide (AO) particles of different sizes. Ninety ceramic premolar crowns were produced with 3Y-TZP frameworks veneered with porcelain. Crowns were randomly divided into three groups, according to the size of the air abrasion AO particles (n = 30): (GC) untreated (control); (G53) 53 µm; (G125) 125 µm. Air abrasion was performed with 0.25 mpa pressure, 10-mm distance, for 10 s. Crowns were adhesively cemented to dentin analog abutments. Specimens were loaded in compression to failure, in 37^o^C distilled water, using a universal testing machine (n = 30). Fractographic analysis was performed using a stereomicroscope and SEM. The roughness of the crown’s inner surface was evaluated using an optical profilometer (n = 10). Fracture load data were statistically analyzed with Weibull analysis and roughness data with Kruskal-Wallis (α = 0.05). GC had the lowest characteristic fracture load (L_0_), while G53 and G125 had higher and statistically similar L_0_ values. The Weibull modulus (m) was similar among groups. The failure modes observed were catastrophic failure and porcelain chipping. There were no differences between the roughness parameters for the experimental groups (p > 0.05). The size of the AO particles did not affect the fracture load and failure mode of 3Y-TZP crowns. Air abrasion with 53 µm and 125 µm particles resulted in a higher fracture load of ceramic crowns than the untreated group while maintaining their reliability and surface characteristics.

## Resumo

O objetivo deste estudo foi investigar o efeito do jateamento com óxido de alumínio (AO) com diferentes tamanhos de partículas no comportamento de fratura de coroas protéticas de zircônia tetragonal estabilizada com 3 mol% de ítria (3Y-TZP). Noventa coroas cerâmicas de pré-molares foram produzidas com infraestruturas de 3Y-TZP recobertas com porcelana. As coroas foram divididas aleatoriamente em três grupos, de acordo com o tamanho das partículas de AO do jateamento (n = 30): (GC) não tratada (controle); (G53) 53 µm; (G125) 125 µm. O jateamento foi realizado com pressão de 0,25 MPa, distância de 10 mm, por 10 s. As coroas foram cimentadas adesivamente em pilares análogos à dentina. As coroas foram testadas em compressão até a falha, em água destilada a 37ºC, usando uma máquina universal de ensaios (n = 30). A análise fractográfica foi realizada usando estereomicroscópio e MEV. A rugosidade da superfície interna das coroas foi avaliada usando um perfilômetro óptico (n = 10). Os dados de carga de fratura foram analisados ​​estatisticamente com análise de Weibull e os dados de rugosidade com Kruskal-Wallis (( = 0,05). GC apresentou a menor carga característica de fratura (L_0_), enquanto G53 e G125 apresentaram valores de L_0_ maiores e estatisticamente semelhantes. O módulo de Weibull (*m*) foi semelhante entre os grupos. Os modos de falha observados foram falha catastrófica e lascamento da porcelana. Não houve diferenças entre os parâmetros de rugosidade para os grupos experimentais (p > 0,05). O tamanho das partículas de AO não afetou a carga de fratura e o modo de falha das coroas 3Y-TZP. O jateamento com partículas de 53 µm e 125 µm resultou em maior carga de fratura das coroas cerâmicas do que o grupo não tratado, mantendo sua confiabilidade e características de superfície.

## Introduction

Zirconia-based ceramics, such as 3 mol.% yttria-stabilized tetragonal zirconia polycrystal (3Y-TZP), gained great popularity in Dentistry due to their high values of fracture strength and toughness, mostly associated to a phase transformation toughening mechanism ^1^. 3Y-TZP frameworks are fabricated using the computer-aided design/computer-assisted manufacturing (CAD/CAM) technology and veneered with more aesthetic feldspathic porcelain or glass-ceramic to produce single-crowns and multi-unit fixed dental prosthesis (FDPs) ^1^ ^-^ ^3^. Although high survival rates have been reported for zirconia-based prostheses, there is great variability among clinical studies ^4^ ^,^ ^5^. These clinical failures are mostly related to processing variables and in-service conditions ^4^ ^-^ ^6^.

Despite the good mechanical properties, 3Y-TZP is acid-resistant and requires a surface treatment, such as air abrasion with aluminum oxide (AO) particles (modified or not by silica), to improve its bond strength to the resin cement ^7^ ^-^ ^9^. Air abrasion with AO particles increases the surface roughness for micromechanical interlocking with the cement, resulting in higher bond strength with 3Y-TZP ceramic compared to untreated surfaces ^7^ ^,^ ^8^ ^,^ ^10^. In addition, AO particles modified by silica are available for air abrasion. The result is a surface covered with a thin and irregular silica layer, which provides both micromechanical retention and chemical bonding among silica and multifunctional agents such as silane ^7^ ^,^ ^10^ ^,^ ^11^. Resin-based adhesives or cements containing a ceramic primer with phosphate-based monomers (i.e. MDP) are also recommended to improve the chemical reaction ^8^ ^,^ ^10^ ^,^ ^11^.

Air abrasion with AO particles is widely used to treat the surface of acid-resistant ceramics because it is an accessible, easy-to-perform, and low-cost option ^12^. Nevertheless, different variables are involved in the air abrasion protocol, including particle type and size; pressure, angle, distance, and duration of the air abrasion process. The parameters chosen to perform the air abrasion protocol can affect the ceramic surface topography, bond strength to the resin cement, and mechanical behavior ^7^ ^,^ ^13^ ^-^ ^17^.

The particle size is an important variable of the air abrasion protocol. The size of AO particles can vary from 25 µm to 150 µm. Previous investigations showed that the particle size has no effect on the bond strength of resin cement to 3Y-TZP ^18^. Yet, results were controversial when the mechanical behavior of 3Y-TZP subjected to air abrasion was investigated. Literature suggested that AO air abrasion improves the flexural strength of 3Y-TZP ^13^ ^-^ ^15^ ^,^ ^19^ ^-^ ^21^. Nevertheless, others claimed that air abrasion with larger particles (120 µm) reduces the flexural strength of 3Y-TZP ^19^ ^,^ ^22^. Moreover, it is important to emphasize that all these investigations used discs and bar-shaped specimens with flat surfaces to evaluate the effect of particle air abrasion on the mechanical behavior of 3Y-TZP. Therefore, the influence of the complex internal geometry of the prosthesis on the surface topography produced by the air abrasion protocol was neglected. The effect of the multi-layer configuration (abutment, resin cement, 3Y-TZP framework, porcelain veneer) on the stress distribution was not considered in these studies as well, which limits the clinical extrapolations.

The main clinical challenge is to define an air abrasion protocol that could improve the resin bond strength of 3Y-TZP without introducing new surface and sub-surface defects that could compromise its mechanical behavior. Thus, the aim of this study is to investigate the failure behavior of 3Y-TZP ceramic crowns subjected to air abrasion with different AO particle sizes. The tested hypotheses are: 1 - air abrasion has no effect on the fracture load, reliability, and roughness of 3Y-TZP crowns; 2 - different particle sizes result in similar values of fracture load, reliability, and roughness of 3Y-TZP crowns.

## Materials and methods

Ninety premolar ceramic crowns were produced, consisting of a 3Y-TZP framework (Vita In-Ceram YZ, Vita Zahnfabrik, Bad Sackingen, BW, Germany) veneered with porcelain (Vita VM9 Dentin Base, 3M3 shade, Vita Zahnfabrik, Bad Sackingen, BW, Germany). Crowns were randomly divided into 3 groups, according to the size of the air abrasion AO particles (n = 30): (GC) untreated (control); (G53) 53 µm; (G125) 125 µm.

### Specimens Preparation

A glass fiber-reinforced epoxy resin material (NEMA G10, International Paper, Hampton, SC, USA) was used to produce the abutments due to its elastic and bonding properties, which are similar to human dentin ^3^ ^,^ ^23^. The dentin analog abutments, simulating a prepared first lower premolar, were designed and milled using a mechanical lathe machine. Abutments had the following dimensions: 6 mm height, rounded shoulder finish line (curvature radius of 0.5 mm), and total occlusal convergence of 12˚ degrees ^3^.

An impression of the abutment was taken using polyvinyl siloxane (Express XT, 3M ESPE, Maplewood, Minnesota, USA). A type IV plaster (Durone, Dentsply/Caulk, Milford, DE, USA) was used to produce the abutment model. Optispray (Sirona Dental Systems, Charlotte, NC, USA) was applied to the abutment model before scanning with InEos Blue optical system (Cerec - Sirona Dental Systems, Charlote, NC, USA). 3Y-TZP ceramic frameworks were designed and milled using the InLab MC XL machine (Cerec - Sirona Dental Systems, Charlotte, NC, USA). Frameworks were sintered according to the manufacturer´s recommendation.

Frameworks were veneered with porcelain by an experienced dental laboratory technician. Two layers of porcelain were applied to obtain the final dimensions of the crown. The sintering cycle was performed according to the manufacturer’s recommendation using Vita Vacumat 40 ceramic furnace (Vita Zahnfabrik, Bad Sackingen, BW, Germany). The final thickness of the crowns was measured using a digital caliper. The final dimensions were 1.5 mm to 2 mm on the axial and occlusal surfaces (framework: 0.5 mm; veneer: 1.0 mm to 1.5 mm).

**Figure 1:**
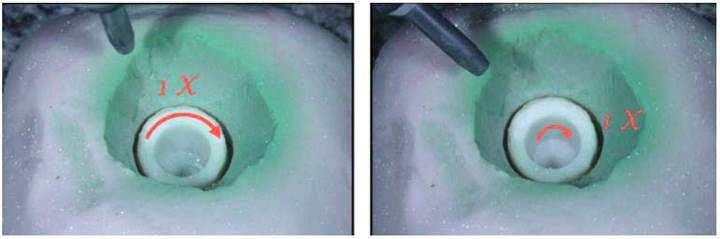
Representative images of the AO air abrasion protocol. The first movement was perpendicular to the inner surface of the crown (reaching the marginal area). The second movement was oblique to the inner surface of the crown (reaching the axial and occlusal surfaces).

For the air abrasion procedure, first, the inner surface of the crown was covered with liquid carbon (Super Filme, Kota, Cotia, SP, Brazil), aiming to control the abraded area. Then, the crown was included in a silicon mold (Zetalabor, Zhermack S. A., Rovigo, Italy) to protect the external surface ^12^. The air pressure was set at 0.25 MPa ^15^ ^,^ ^17^ and a spacer was used to assure a 10 mm distance between the crown margin and the tip of the air abrasion device. Two movements were performed in a clockwise direction. The first movement was perpendicular to the inner surface of the crown (reaching the marginal area) and the second movement was oblique to the inner surface of the crown (reaching the axial and occlusal surfaces) (Figure 1). The total air abrasion time was, approximately, 10 seconds. Air abrasion was performed by a single calibrated operator using the Microetcher II equipment (Danville Engineering, San Ramon, CA, USA) attached to a pressure regulator (Model FRAC-10A, Fambras, SP, Brazil).

All crowns were sonically cleaned in a bath with 96% alcohol for 3 minutes. Before cementation, the bonding surface of the abutment was etched with 10% hydrofluoric acid for 1 minute (Cond AC Porcelana, FGM, Joinville, SC, Brazil), washed with water for 30 seconds, and air-dried. G10 acid-etching was performed to expose the glass fibers and to create micro-mechanical retentions to improve the bond strength ^3^ ^,^ ^23^. A silane bonding agent was applied to the abutment surface and let evaporate for 1 minute (Prosil, FGM, Joinville, SC, Brazil). The cementation protocol followed the manufacturer´s instructions. The adhesive system (ED Primer A + B, Kuraray, Tokyo, Japan) was applied on the abutment surface followed by the resin cement (Panavia F, Kuraray, Tokyo, Japan). Crowns were placed on the abutments with digital pressure. A static load of 2 kg was applied for 1 minute to the occlusal surface of the crowns using a cementation device. Each side of the crown (buccal, lingual, mesial, and distal) was light-cured for 20 seconds (CL-K50, Kondortech, São Carlos, SP, Brazil). After complete polymerization, an oxygen inhibitor (Panavia F Oxyguard II, Kuraray, Tokyo, Japan) was applied to the cervical region for 3 minutes. The cemented crowns were stored in 37ºC distilled water for 48 hours before the mechanical tests.

### Fracture Load Test

The test was performed in a 37ºC distilled water bath using a universal testing machine (EMIC DL 2000 Brasil, São José dos Pinhais, PR, Brazil). The load was applied in the center of the occlusal surface using a spherical stainless steel piston of 6 mm in diameter, at a cross-head speed of 0.5 mm/min, until the fracture of the veneer porcelain or the zirconia framework ^2^.

The maximum fracture load values were recorded and Weibull statistics was used to determine the Weibull modulus (*m*) and characteristic fracture load (L_0_). The 95% confidence intervals for *m* and L_0_ were calculated using maximum likelihood estimate (MLE) with Weibull++ statistical software (ReliaSoft). A sample size of 30 is recommended to perform an accurate Weibull analysis.

The failure mode was classified as catastrophic failure (fracture of the porcelain and the framework) or chipping (only porcelain fracture). Fractographic analysis was performed using a stereomicroscope (SZ61, Olympus Corp., Tokyo, Japan). The fracture surface of representative crowns of each group was coated with gold-palladium and examined under scanning electron microscopy (SEM) (Model Vega 3, TESCAN, Brno, Czech Republic, Czechoslovakia) to investigate the fracture origin and direction of crack propagation ^6^. The chi-square test was used to verify the association between failure mode and the experimental group (( = 0.05).

### Roughness Analysis

Before cementation, 10 crowns from each group were randomly selected for roughness analysis using an optical profilometer (Proscan 2100, Scantron, Venture Way, Taunton, UK). It was possible to obtain the roughness parameters only from the inner occlusal region of the crowns, since a flat surface is required for the analysis.

The device was calibrated with a measurement filter of 0.25 mm (cut-off). Proscan 2000 program was used for the analyses. Measurements were performed in a 2-mm^2^ area. In each crown, 100 measurements were performed, 100 on the X axis and 100 on the Y axis, to determine the parameters: (Ra) arithmetic mean roughness; (Rz) average maximum height of the profile; (Rmax) maximum roughness depth; (Rq) root mean square deviation of the evaluated profile.

For surface roughness, a statistical software (G*Power) was used to calculate the sample size (n = 10) using the following parameters: effect size = 0.62, ( = 0.50; power = 0.80; the number of groups = 3. The effect size was estimated using roughness data collected in a pilot study. The roughness data were statistically analyzed using Kruskal-Wallis non-parametric test, considering that data failed the normality and homoscedasticity tests (α = 0.05).

## Results

**Figure 2:**
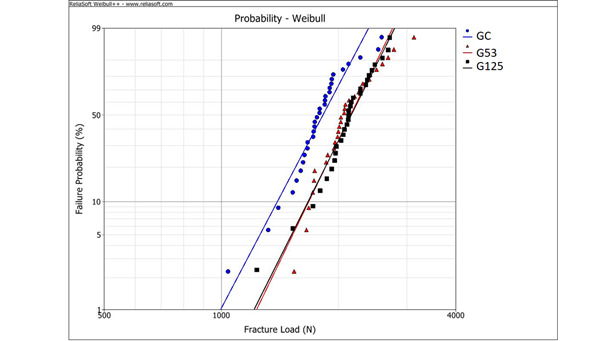
Weibull graph showing fracture load data for the experimental groups.

Weibull modulus (*m*), characteristic fracture load (L_0_) and fracture load for a 5% probability of failure (L_5%_) for the experimental groups are presented in Table 1 and Figure 2. For the parameters *m* and L_0,_ a statistical difference was considered when the values of the 95% confidence intervals of the experimental groups do not overlap. G53 and G125 had statistically similar and higher values of L_0_ than GC. The *m*-value was similar among the groups.

**Table 1:** Weibull modulus values (*m*), characteristic fracture load (L_0_) and respective 95% confidence intervals (95% CI) for the experimental groups, and fracture load for a 5% probability of failure (L_5%_). Frequency of each failure mode by group.

Groups	Weibull modulus					Failure mode (%)**	
m*	CI (95%) - *m*	L_0_ (N)*	CI (95%) - L_0_ (N)	L_5%_ (N)	Catastrophic	Chipping
GC	7.0 a	5.5; 8.9	1920 b	1813-2034	1256	56.6	43.3
G53	7.6 a	6.0; 9.6	2253 a	2127-2387	1525	53.3	46.6
G125	7.4 a	5.5; 9.8	2266 a	2151-2387	1514	58.6	41.4

*Values followed by the same letter in the same column are statistically similar.

**Chi-square test, non-significant (p=0.918).

**Figure 3:**
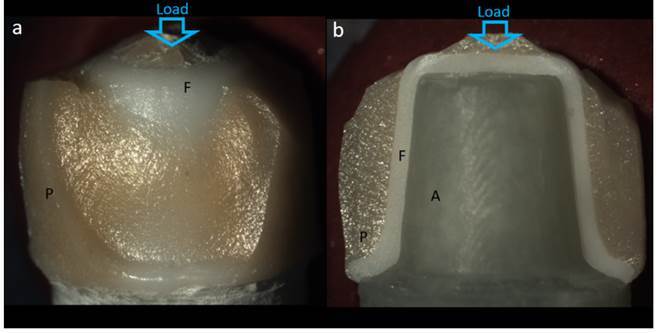
Representative images of the failure modes observed for the ceramic crowns tested in compression: (A) chipping, and (B) catastrophic fracture. A - abutment; F - framework; P - porcelain.

**Figure 4:**
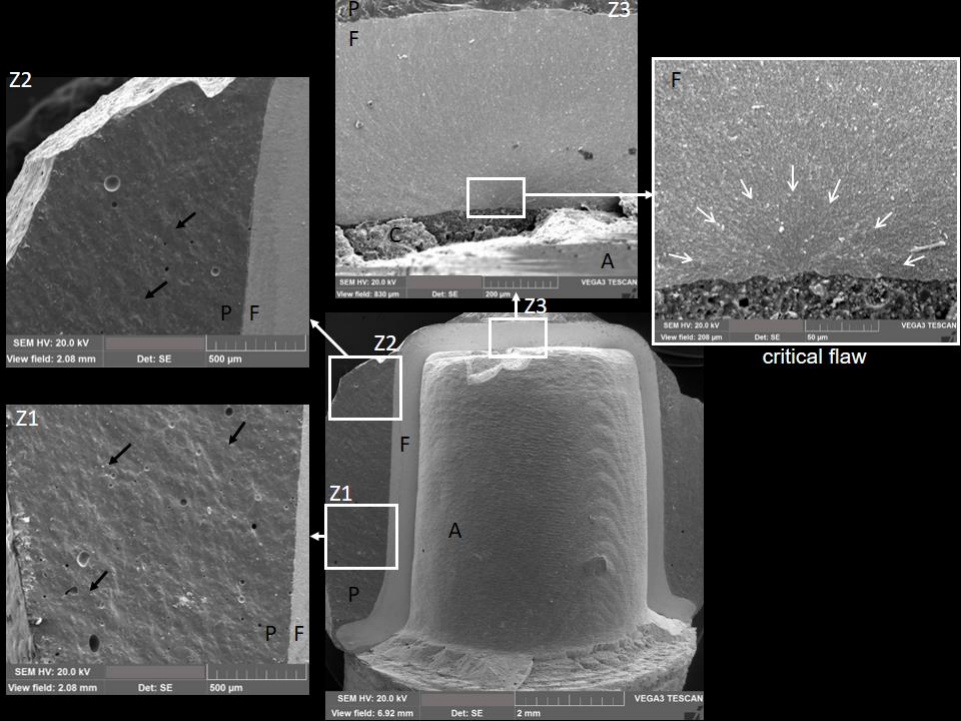
Map of the fracture surface of a 3Y-TZP crown from group G53 (crown shown in Figure 3B). It is possible to identify fractographic features such as wake hackles, in the porcelain (P) layer at zones 1 (Z1) and 2 (Z2), indicating the direction of crack propagation (black arrows). Hackle lines in the occlusal area, at zone 3 (Z3), point to the critical flaw located at the inner surface of the zirconia framework (F) (delimited by white arrows). Surface characteristics (symmetrical groves) at the critical flaw suggest the presence of grinding damage. A - abutment; C - resin cement.

The frequency of each failure mode for each experimental group is described in Table 1. There was no association between failure mode and the experimental group (p = 0.918). Groups showed both catastrophic and chipping failure modes. Chipping involved the porcelain veneer, and the fracture origin was at the contact zone between the loading piston and the porcelain, propagating towards the cervical area of the crown and exposing the zirconia framework, as observed in Figure 3a. Catastrophic fracture involved both porcelain and zirconia framework (Figure 3b), and the flaw origin was mostly located at the inner occlusal surface of the zirconia framework (radial crack) (Figure 4). Catastrophic fracture initiating from the contact damage at the occlusal area of the crown was observed for only a few specimens.

**Figure 5:**
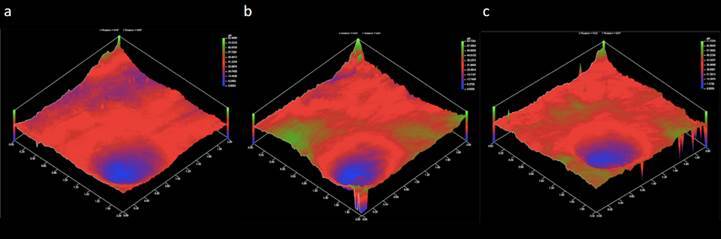
Profilometry images of the inner occlusal surface of a crown from groups GC (A); G53 (B); and G125 (C).

For the roughness parameters (Ra, Rmax, Rz and Rq), no statistical difference was observed between the groups (p > 0.05) (Table 2). Representative images of the crown’s inner occlusal surface are shown in Figure 5.

**Table 2:** Median and interquartile range (Q_1_; Q_3_) values of roughness parameters Ra, Rz, Rmax and Rq for the experimental groups (µm).

	Roughness Parameters*			
	Ra	Rz	Rmax	Rq
GC	6.7 a (6.5; 7.1)	13.9 a (13.0; 14.5)	21.3 a (20.2; 22.8)	8.0 a (7.8; 8.3)
G53	7.0 a (6.5; 7.2)	12.0 a (11.9; 14.3)	18.6 a (17.6; 20.9)	8.1 a (7.5; 8.9)
G125	6.9 a (6.5; 7.8)	12.9 a (12.3; 13.7)	19.0 a (18.0; 21.1)	8.1 a (7.5; 9.1)
p**	0.921	0.194	0.246	0.982

*Values followed by the same letter in the same column are statistically similar (p>0.05)

**p-value calculated for each parameter with Kruskal-Wallis test ((=0.05)

## Discussion

Air abrasion with 50 µm and 125 µm AO particles increased the fracture load of 3Y-TZP crowns, but had no influence on their reliability and roughness of the inner occlusal surface, partially accepting the first study hypothesis. This finding is consistent with other studies that reported an increase in the mechanical strength of 3Y-TZP subjected to particle air abrasion ^13^ ^-^ ^15^ ^,^ ^20^ ^,^ ^24^.

According to the literature, mechanical aggression caused by particle air abrasion could introduce defects on the 3Y-TZP surface, but the nature and characteristics of these defects are not fully understood. Particles are moved in an airflow to impact the inner surface of the ceramic restoration, generating compressive stresses and causing a plastic deformation of the affected surface. Depending on the intensity of the AO particle’s impact, tensile stresses are generated around the plastic deformation areas, introducing micro-cracks parallel and/or perpendicular to the surface ^16^ ^,^ ^22^ ^,^ ^25^. Nevertheless, these defects can be stabilized by compressive stresses around the crack tip resulting from the volumetric expansion (approximately 3 to 5%) associated with the tetragonal (t) to monoclinic (m) zirconia phase transformation; and also by the compressive stresses induced by the plastic deformation of the material ^1^ ^,^ ^7^. These mechanisms could partially explain the greater fracture load values observed in the present study for 3Y-TZP crowns subjected to particle air abrasion.

Another important issue is the fact that CAD/CAM milling of 3Y-TZP restorations could introduce defects that are more critical to the material´s mechanical properties than particle air abrasion itself ^6^ ^,^ ^15^ ^,^ ^19^. The fractographic analysis of different types of 3Y-TZP subjected to air abrasion found that the fracture origins were either associated with the milling processes (cutting direction of the blocks) or with material’s fabrication and sintering flaws; but not resulting from the air abrasion processes. In Figure 4, in the image delimited by the white box (critical flaw), it is possible to observe symmetrical groves in the surface close to the fracture origin. Fractographic studies indicated that these types of grooves could be produced by machining tools, such as the ones used for CAD/CAM milling ^6^ ^,^ ^15^. Moreover, a 15% to 30% increase in flexural strength has been reported for different 3Y-TZP ceramics subjected to air abrasion with low pressure (0.25 MPa) using alumina particles modified by silica (30 µm). The authors concluded that particle air abrasion had a smoothing effect of the defects produced by the machining process in the 3Y-TZP surface ^15^.

The size of the aluminum oxide particles used in the air abrasion protocol did not influence the fracture load, reliability, and roughness of 3Y-TZP crowns, accepting the study’s second hypothesis. The maximum fracture load values were similar for 3Y-TZP crowns subjected to air abrasion with small (53 µm) and large (125 µm) particles, corroborating with the literature findings ^14^ ^,^ ^24^.

The size of the aluminum oxide particles used in the air abrasion protocol did not influence the fracture load, reliability, and roughness of 3Y-TZP crowns, accepting the study’s second hypothesis. The maximum fracture load values were similar for 3Y-TZP crowns subjected to air abrasion with small (53 µm) and large (125 µm) particles, corroborating with the literature findings ^14^ ^,^ ^24^.

In the present study, air abrasion was not able to alter, significantly, the occlusal inner surface topography of the crowns. Yet, a previous study evaluated the surface roughness of untreated (polished) and air-abraded (50 µm AO particles) 3Y-TZP specimens and observed greater values for the treated group ^8^. Moreover, an investigation reported greater surface roughness of 3Y-TZP specimens with increasing particle size ^18^, which was also not observed in the present study. First, roughness data should be interpreted considering that the roughness parameters were obtained only from the inner occlusal region of the crowns, since a flat surface was required for the profilometry analysis. The topography of this area was not significantly altered by air abrasion with the different AO particle sizes mainly due to the greater distance from the air abrasion device tip and low pressure used in the protocol (0.25 MPa). A 10-mm distance was calculated from the crown margin to the air abrasion device tip. Thus, the distance from the occlusal region was, approximately, 15 mm. Moreover, due to the complex internal geometry of the crown, it is not possible to guarantee that all regions will be homogeneously particle air abraded. Nevertheless, this protocol reproduces the one applied clinically, being different from the uniform particle air abrasion of polished and flat ceramic surfaces used in most in vitro investigations.

Crowns had similar failure modes: 41 to 47% of catastrophic fractures, and 53 to 59% of porcelain chipping, which are failure modes found clinically ^1^ ^,^ ^4^ ^-^ ^6^. Radial cracks located at the inner surface of the 3Y-TZP frameworks, in the opposite side of the load application site, resulted in catastrophic failure of the crowns. Contact damage resulted in chipping failures and a few catastrophic fractures as well ^1^ ^,^ ^4^ ^-^ ^6^. In addition, the fact that there was no difference in Weibull modulus among the experimental groups also suggests a similar population (distribution and size) of defects related to the crown failure.

To reproduce the clinical scenario, ceramic crowns were produced following the manufacturer's instructions: 3Y-TZP frameworks were milled using CAD/CAM system, veneered with porcelain, and crowns were cemented adhesively with MDP resin cement onto a dentin analog abutment ^2^ ^,^ ^3^ ^,^ ^12^ ^,^ ^23^. The air abrasion protocol was developed according to the parameters used by laboratory technicians and dentists and recommended by the literature ^7^. Conventional AO particles were chosen in the present study considering their availability and cost/benefit ratio. Moreover, a study found similar bond strength values between resin cement and 3Y-TZP treated with conventional or silica-coated AO particles after aging for 1 and 2 years ^11^. However, the results of this study are valid for the second 3Y-TZP generation and clinical extrapolations should consider the influence of cyclic fatigue on the fracture behavior of ceramic crowns ^1^ ^,^ ^15^ ^,^ ^16^. Developing methods to quantify the tetragonal-to-monoclinic phase transformation in the fracture origin, to measure residual stresses of crown-shaped specimens, and to characterize the topography at all internal regions of the crowns are recommended for future investigations. In the present study, a fracture load test was used considering the lack of information on the mechanical behavior of particle-abraded 3Y-TZP crown-shaped specimens. Fast fracture tests are efficient tools to collect data and to screen variables for further fatigue evaluations ^3^.

Within the limitations of this in vitro study, it can be concluded that aluminum oxide air-abraded 3Y-TZP crowns had greater maximum fracture load values than untreated crowns, but similar reliability and surface roughness. Additionally, the particle size had no influence on the maximum fracture load, reliability, and roughness of 3Y-TZP crowns. Therefore, the air abrasion protocols evaluated in the present study - 0.25 MPa air pressure, 10 mm distance from the crown margin, total abrasion time of 10 s, AO particles of 53 µm or 125 µm - could be recommended for clinical use, as they were not able to introduce new surface and sub-surface defects that could compromise the mechanical behavior of 3Y-TZP crowns.
